# Mitigating Hydroxychloroquine-Induced Oxidative Liver Damage: The Roles of Adenosine Triphosphate, Liv-52, and Their Combination in Rats

**DOI:** 10.3390/ijms27010421

**Published:** 2025-12-31

**Authors:** Meryem Yalvac Kandefer, Esra Tuba Sezgin, Bahadir Suleyman, Ferda Keskin Cimen, Fulya Memiş, Mine Gulaboglu, Halis Suleyman

**Affiliations:** 1 Department of Internal Medicine, Mengucek Gazi Education and Research Hospital, Erzincan Binali Yıldırım University, Erzincan 24100, Turkey; meryemyalvac@hotmail.com (M.Y.K.); fozel57@gmail.com (F.M.); 2 Anesthesia Program, Vocational School of Health Services, Erzincan Binali Yıldırım University, Erzincan 24100, Turkey; esra.demir@erzincan.edu.tr; 3 Department of Pharmacology, Faculty of Medicine, Erzincan Binali Yıldırım University, Erzincan 24100, Turkey; bahadirsuleyman@yandex.com (B.S.); halis.suleyman@gmail.com (H.S.); 4 Department of Medical Pathology, Faculty of Medicine, Erzincan Binali Yıldırım University, Erzincan 24100, Turkey; 5 Department of Biochemistry, Faculty of Pharmacy, Ataturk University, Erzurum 25240, Turkey; minegulaboglu@atauni.edu.tr

**Keywords:** ATP, HCQ, Liv-52, liver damage, oxidative stress, rats

## Abstract

Hydroxychloroquine (HCQ), originally developed as an antimalarial agent, has been associated with hepatotoxic effects in experimental and clinical settings. Our study was designed to evaluate the effects of this agent on liver toxicity and to understand the protective roles of adenosine triphosphate (ATP), Liver-52 (Liv-52), and their combination. Male Wistar rats (250–280 g) were randomly assigned to five groups (n = 6): healthy control (C), HCQ only (H), ATP plus HCQ (AH), Liv-52 plus HCQ (LH), and ATP–Liv-52 plus HCQ (ALH). ATP (4 mg/kg) was administered intraperitoneally once daily, whereas Liv-52 (20 mg/kg) was administered orally via gavage. One hour later, all groups except C received HCQ (120 mg/kg, orally, twice daily). All treatments were continued for seven consecutive days. At the end of the experiment, serum aspartate aminotransferase (AST) and alanine aminotransferase (ALT) levels were measured, and liver tissues were analyzed for malondialdehyde (MDA), total glutathione (GSH), superoxide dismutase (SOD), and catalase (CAT) activities, along with histopathological evaluation. HCQ administration significantly increased oxidative stress, as evidenced by elevated MDA levels (*p* < 0.01) and reduced antioxidant parameters, including GSH, SOD, and CAT (*p* < 0.05), accompanied by prominent histopathological damage. Treatment with ATP or Liv-52 markedly ameliorated these alterations by decreasing MDA and restoring antioxidant markers. The combination treatment was observed to exhibit the most pronounced protective effect; it significantly reduced MDA levels, improved GSH, SOD, and CAT levels more effectively, and produced significant decreases in AST and ALT values (*p* < 0.05).

## 1. Introduction

HCQ is a 4-aminoquinoline derivative drug that was initially used as an antimalarial agent [1]. In subsequent years, with the elucidation of its anti-inflammatory properties, it began to be utilized in the treatment of various rheumatic diseases, most notably rheumatoid arthritis [2,3]. Authorized for use in the United States in 1994, the indications for HCQ have expanded over time, and it was utilized in the treatment of COVID-19 during the pandemic due to its antiviral potential [2,4]. While it generally exhibits a safe profile, various side effects associated with HCQ use have been reported in the literature [5]. Particularly during the COVID-19 pandemic, serious adverse effects such as hepatotoxicity, cardiotoxicity, severe skin reactions, and suicidality have garnered attention [6]. Indeed, a significant increase, up to ten times, in serum transaminase levels was observed following HCQ administration in a patient diagnosed with COVID-19, highlighting the hepatotoxic potential of this drug [7]. Furthermore, acute severe liver damage related to HCQ has also been confirmed by liver biopsy [8]. Current findings suggest that HCQ-related toxicity may occur through a multifactorial mechanism, including oxidative stress [9]. Previous studies have shown that HCQ can affect various signaling pathways associated with oxidative stress, including PI3K/Akt, Nrf2, and NF-κB. These pathways play critical roles in regulating antioxidant defense and inflammatory responses. Dysregulation of these mechanisms may lead to excessive production of reactive oxygen species (ROS), increased lipid peroxidation, reduced antioxidant enzyme levels, and ultimately hepatocellular damage [10]. Additionally, it has been shown that HCQ reduces intracellular ATP [11].

ATP, a nucleotide comprising adenine, ribose sugar, and three phosphate groups, plays a central role in cellular energy metabolism [12]. Intracellular ATP is involved in the synthesis of antioxidants that participate in the elimination of ROS, providing the necessary energy for these processes [13,14]. The literature indicates that ATP deficiency may trigger cellular necrosis [15,16]. This information suggests that antioxidant therapies could be beneficial in preventing HCQ-induced oxidative liver damage. Liv-52, a multi-herbal tablet formulation composed of extracts of *Cichorium intybus*, *Cassia occidentalis*, *Capparis spinosa*, *Solanum nigrum*, *Terminalia arjuna*, *Achillea millefolium* and *Tamarix gallica* is known for its effects in preventing cytotoxicity induced by oxidative stress. Liv-52 inhibits mitochondrial β-oxidation of fatty acids due to its capacity to reduce lipid peroxidation (LPO), thereby decreasing MDA levels and preventing GSH depletion [17,18]. All of the aforementioned findings suggest that both ATP and Liv-52 may have protective potential against the possible oxidative liver damage induced by HCQ. However, there have been no studies investigating the protective effects of ATP, Liv-52, or their combination against HCQ hepatotoxicity. In this regard, the aim of our study is to evaluate the toxic effects of HCQ on liver tissue in rats and to investigate the possible hepatoprotective effects of ATP, Liv-52, and the combination of these two agents.

## 2. Results

### 2.1. Biochemical Findings

#### 2.1.1. The Outcomes of the MDA and tGSH Assays in Liver Tissue

**Figure 1:**
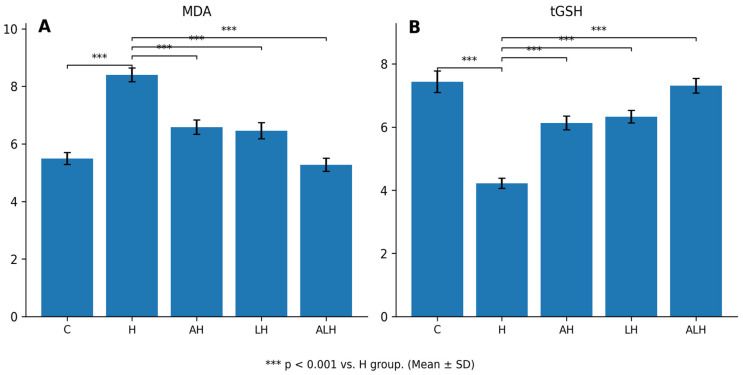
(**A**,**B**) Effects of HCQ, ATP, and Liv-52 on oxidative stress parameters (MDA and tGSH) in rat liver tissue. Values are expressed as mean ± SD (n = 6). Statistical analyses were performed using one-way ANOVA followed by Tukey’s HSD post hoc test. *** *p* < 0.001 vs. H group. Abbreviations: ATP: adenosine triphosphate; C: healthy group; H: hydroxychloroquine alone group; AH: ATP + hydroxychloroquine group; LH: Liv-52 + hydroxychloroquine group; ALH: ATP + Liv-52 + hydroxychloroquine group; MDA: malondialdehyde; tGSH: total glutathione.

HCQ administration caused marked oxidative injury by increasing MDA levels in liver tissue by 57% (Figure 1A). The difference in MDA levels between the healthy and HCQ groups was statistically significant (*p* < 0.001). This HCQ-induced increase was significantly suppressed by both ATP (*p* < 0.001) and Liv-52 (*p* < 0.001) treatments. In contrast, MDA levels in the ALH group were decreased by 37%. 

HCQ was also found to reduce tGSH levels in liver tissue by 43% (Figure 1B). The difference in tGSH levels between the healthy and HCQ groups was statistically significant (*p* < 0.001). Administration of ATP (*p* < 0.001) and Liv-52 (*p* < 0.001) significantly attenuated the HCQ-induced decrease in tGSH levels. Conversely, a 73% increase in tGSH levels was observed in the ALH group.

#### 2.1.2. The Outcomes of the SOD and CAT Assays in Liver Tissue

**Figure 2:**
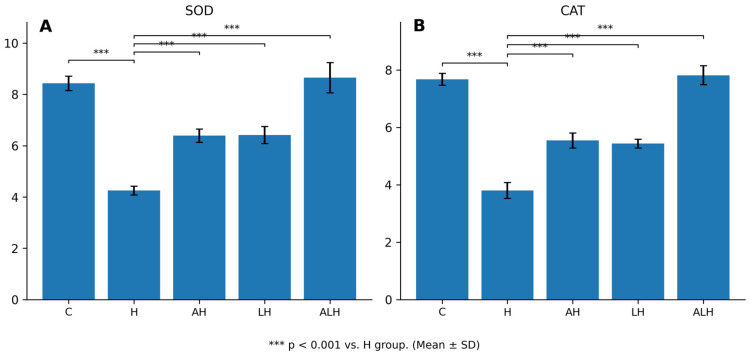
(**A**,**B**) Effects of HCQ, ATP, and Liv-52 on oxidative stress parameters (SOD, CAT) in rat liver tissue. Values are expressed as mean ± SD (n = 6). Statistical analyses were performed using one-way ANOVA followed by Tukey’s HSD post hoc test. *** *p* < 0.001 vs. H group. Abbreviations: ATP: adenosine triphosphate; C: healthy group; H: hydroxychloroquine alone group; AH: ATP + hydroxychloroquine group; LH: Liv-52 + hydroxychloroquine group; ALH: ATP + Liv-52 + hydroxychloroquine group; SOD: superoxide dismutase; CAT: catalase.

HCQ administration caused a pronounced antioxidant impairment by reducing SOD and CAT activities in liver tissue by 50% (Figure 2A,B). Accordingly, SOD (*p* < 0.001) and CAT (*p* < 0.001) activities in the HCQ-treated group were significantly lower than those observed in the healthy group. This HCQ-induced reduction in antioxidant enzyme activities was significantly attenuated by both ATP (SOD and CAT, respectively, *p* < 0.001; *p* < 0.001) and Liv-52 (SOD and CAT, respectively, *p* < 0.001; *p* < 0.001) administrations. In contrast, ALH treatment increased SOD and CAT activities by 100%.

#### 2.1.3. The Outcomes of the Blood Serum ALT and AST Assays

**Figure 3:**
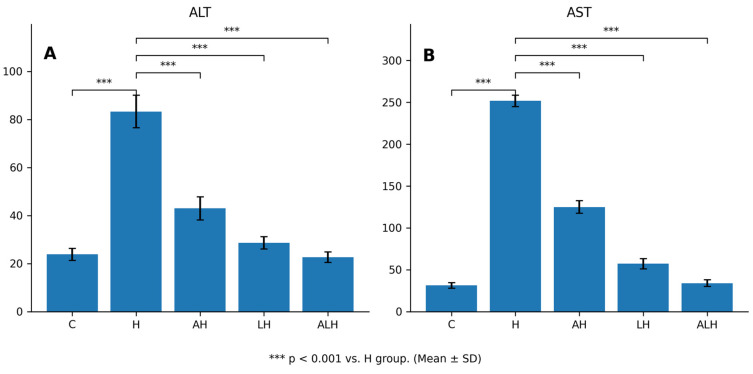
(**A**,**B**) Effects of HCQ, ATP, and Liv-52 on liver enzyme activities (ALT and AST) in rat liver tissue. Values are expressed as mean ± SD (n = 6). Statistical analyses were performed using Welch ANOVA followed by the Games–Howell post hoc test. *** *p* < 0.001 vs. H group. Abbreviations: ATP: adenosine triphosphate; C: healthy group; H: hydroxychloroquine alone group; AH: ATP + hydroxychloroquine group; LH: Liv-52 + hydroxychloroquine group; ALH: ATP + Liv-52 + hydroxychloroquine group; ALT: alanine transaminase; AST: aspartate transaminase.

HCQ administration caused marked hepatocellular injury by increasing serum ALT activity by 260% (Figure 3A). Accordingly, ALT activity in the HCQ-treated group was significantly higher than that observed in the healthy group (*p* < 0.001). This HCQ-induced elevation in ALT activity was significantly attenuated by ATP administration (*p* < 0.001). Notably, Liv-52 was more effective than ATP in preventing the HCQ-associated increase in ALT activity, and a significant difference was observed between the ATP and Liv-52 groups (*p* = 0.002). In contrast, ALH treatment reduced ALT levels by 73%.

HCQ treatment also resulted in a severe increase in serum AST activity, with AST levels elevated by 700% compared to the healthy group (Figure 3B). Consistently, AST activity in the HCQ group was significantly higher than that in the healthy group (*p* < 0.001). ATP significantly reduced the HCQ-induced increase in AST activity (*p* < 0.001), whereas Liv-52 exerted a more pronounced protective effect (*p* < 0.001). A significant difference in AST activity was detected between the ATP and Liv-52 groups (*p* < 0.001). Furthermore, the combined administration of ATP and Liv-52 prevented approximately 90% of the HCQ-induced elevation in AST activity.

### 2.2. Histopathological Findings

**Figure 4:**
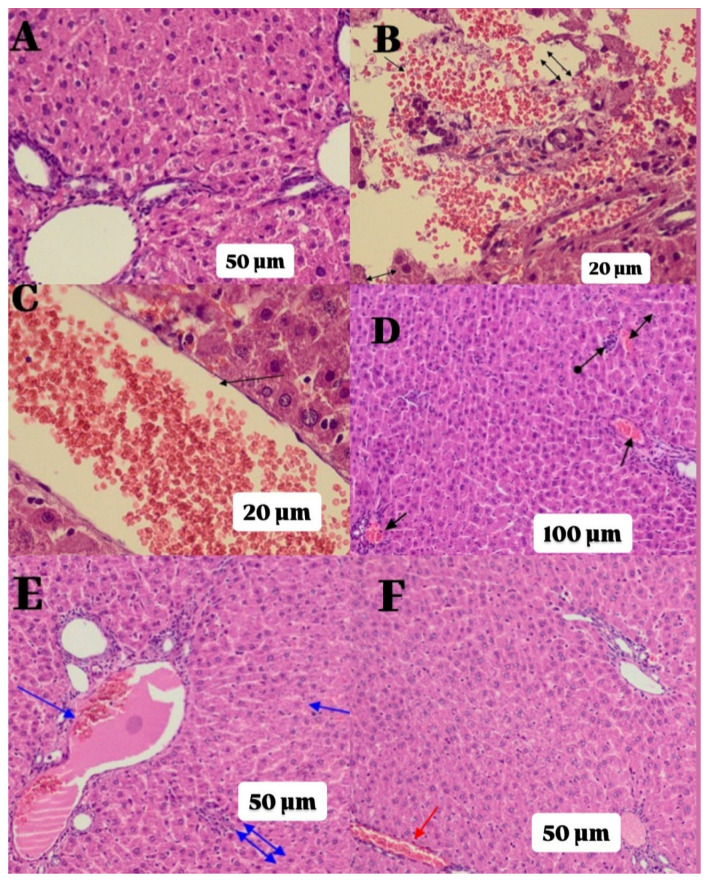
Histopathological evaluation of liver tissue in experimental groups (H&E staining). Representative photomicrographs from each group (n = 6). (**A**) Healthy group (C): normal hepatic parenchyma and portal area (×200). (**B**) HCQ-alone group (H): severe hepatic injury characterized by parenchymal destruction (double-headed arrow), hemorrhage (single-headed arrow), and edema (double arrow) (×400). (**C**) HCQ-only group (H): markedly dilated and congested blood vessel (×400). (**D**) ATP + HCQ group (AH): moderate hepatic damage with dilated congested blood vessel (single arrow), hemorrhage (double-headed arrow), and parenchymal lymphocyte infiltration (double arrow) (×200). (**E**) Liv-52 + HCQ group (LH): moderate hepatic damage characterized by dilated congested blood vessel (single arrow), hemorrhage (double-headed arrow), and parenchymal lymphocyte infiltration (double arrow) (×200). (**F**) ATP + Liv-52 + HCQ combination group (ALH): near-normal liver tissue morphology with only mild vascular congestion (red arrow) (×200).

Table 1 summarizes the histopathological grading results from rat liver samples and the statistical comparisons based on *p*-values. As observed in Figure 4A, the C group exhibited healthy normal portal areas and parenchyma in the liver tissue. The liver tissue of the group treated solely with HCQ displayed severe parenchymal destruction, hemorrhage, edema, and significant dilation of congested blood vessels, indicating severe damage (Grade-3; Figure 4B,C). Some improvement was noted in the liver tissue of the AH group. However, moderate damage (Grade-2), characterized by dilated congested blood vessels, hemorrhage, and lymphocyte infiltration in the parenchyma, was still observed (Figure 4D). The liver tissue of the LH group displayed similar morphology to that of the ATP group, exhibiting moderate damage (Grade-2) with dilated congested blood vessels, hemorrhage, and lymphocyte infiltration (Figure 4E). As shown in Figure 4F, the best level of recovery from HCQ-induced tissue damage was observed in the ALH group, where ATP and Liv-52 were administered in combination. In this group, the liver tissue appeared close to C group with only a slight dilation of congested blood vessels.

## 3. Discussion

This study assessed the protective effects of ATP, Liv-52, and their combination against HCQ-induced oxidative liver injury. HCQ markedly elevated hepatic MDA levels and reduced tGSH, SOD, and CAT, while also increasing serum transaminase levels. Originally developed as an antimalarial drug, HCQ is now used to treat immune-mediated rheumatic disorders such as systemic lupus erythematosus and rheumatoid arthritis [5,19]. Despite all of these therapeutic effects, HCQ has been associated with side effects such as cardiomyopathy, neuromyopathy, and hematological abnormalities [20,21]. In comparison to these adverse reactions, hepatotoxicity associated with HCQ has been reported less frequently, and its pathophysiology is not fully understood. The literature contains mostly case reports regarding this issue [7,22,23]. Additionally, a limited number of reports have described fulminant liver failure [24]. Studies have reported that oxidative stress is one of the important factors in the pathogenesis of HCQ-related toxicity [25,26,27]. As known, oxidative stress is characterized by an increase in ROS production and a decrease in antioxidant defense mechanisms [28]. Various studies have shown that HCQ administration leads to excessive ROS production in tissues, resulting in a significant increase in MDA levels, a byproduct of LPO [25,26]. Topak et al. reported that oral administration of HCQ in rats triggered oxidative stress by increasing MDA levels and adversely affected the fracture healing process [25]. Similarly, Abou-khzam, F. and B. reported that oral administration of HCQ significantly elevated MDA levels in liver tissue of rats [29]. However, there are also findings in the literature suggesting that HCQ has inhibitory effects on oxidative stress, supports antioxidant defense systems, and facilitates the repair of tissue damage [30,31]. The biochemical data from our study demonstrated that MDA levels were significantly increased in the liver tissues of animals treated with HCQ. These results align with some previous studies emphasizing the oxidizing effects of HCQ [25,26,29], but they contradict other experimental studies that reported HCQ’s ability to suppress oxidative stress [30,31]. It is believed that the discrepancies in these findings may stem from methodological variables such as dosage, duration of administration, animal models, or the type of tissue evaluated. In the current literature, various studies have demonstrated that the use of antioxidant molecules has yielded positive results in mitigating oxidative stress induced by HCQ in tissues [29,32]. The findings of our study also support this observation. In animals treated with HCQ, a significant increase in oxidant capacity was observed; conversely, essential antioxidant defense mechanisms such as tGSH, SOD, and CAT were found to be significantly reduced. GSH is one of the most important antioxidant molecules in the intracellular environment, composed of glutamate, cysteine, and glycine [33]. Particularly in drug-induced liver injury, insufficient hepatic GSH levels can lead to the formation of protein adducts in the mitochondria by toxic drug metabolites, resulting in mitochondrial dysfunction and cell death [34]. Indeed, the literature indicates that HCQ depletes GSH stores in CD4+ T cells, increases mitochondrial superoxide production, and inhibits cell proliferation [35]. In light of this information, the decrease in tGSH levels observed in our study due to HCQ supports the potential for hepatocellular damage. Additionally, it was noted that in parallel with the decrease in tGSH, the activities of SOD and CAT enzymes were also reduced in the HCQ-treated group. These enzymes form a primary line of defense against oxidative stress; their activities are evaluated as diagnostic and prognostic biomarkers in various diseases [36]. Topak et al. reported that oral administration of HCQ in rats decreased SOD and CAT activity, negatively affecting fracture healing [25]. These findings are consistent with the data from our study. On the other hand, some literature sources have also reported results indicating that HCQ is associated with higher GSH levels and increased SOD/CAT activity, thereby mitigating oxidative damage [31,37]. To evaluate HCQ-induced hepatocellular damage, blood serum levels of ALT and AST were measured from samples collected from the tail veins of the animals. Increases in these enzymes in serum are considered biochemical markers of hepatocyte damage and necrosis [38]. In the literature, toxic effects on the liver in patients undergoing HCQ treatment have been documented with elevated serum AST and ALT levels, supported by various case reports [7,21,39]. Additionally, Sayed et al. reported significant increases in serum ALT and AST levels along with the development of hepatocellular degeneration in a study they conducted on a fish species exposed to HCQ [10]. The findings from our study are consistent with these data, showing that in the HCQ-treated group, serum ALT and AST levels were significantly higher compared to the healthy control group. A commonly used biomarker for assessing liver function, the AST/ALT ratio (De Ritis ratio), also holds clinical importance in differentiating hepatobiliary disorders [40]. In our study, this ratio was calculated to be approximately 3.0 in the HCQ group, indicating AST dominance and serving as a finding supporting HCQ-induced hepatotoxicity. It is known that HCQ increases cellular ATP consumption and accelerates proton flow across the lysosomal membrane [15]. ATP, ADP, and adenosine play crucial roles in the biotransformation and metabolism of xenobiotics in hepatocytes. They also function as extracellular signaling molecules involved in regulating various physiological and pathophysiological processes [41]. A disruption of the proton gradient that directs ATP production in the mitochondria can trigger necrotic or apoptotic cell death in the liver [42]. However, some literature sources indicate that ATP is an essential energy source for the synthesis of ROS-scavenging antioxidants; externally supplied ATP supplementation has been shown to protect organs against oxidative damage and improve their functions [43,44]. Furthermore, extracellular ATP mediates its effects primarily through purinergic receptors, which are pivotal in regulating oxidative stress responses and modulating inflammation within hepatic tissues [45]. The findings of our study align with this literature. In animals treated with HCQ that received ATP supplementation, the increase in MDA levels in the liver tissue was significantly suppressed, and the decline in tGSH levels along with SOD and CAT enzyme activities was prevented. Furthermore, the increase in serum AST and ALT levels was also significantly hindered by ATP treatment. These results highlight the supportive effects of ATP on the antioxidant defense system and its role in reducing HCQ-induced hepatocyte damage. Liv-52 is a polyherbal Ayurvedic formulation that has been shown to have protective effects against hepatotoxicity induced by chemical agents, both in clinical and preclinical studies [17,46]. This preparation is indicated for the management of acute liver injury, viral hepatitis, early-stage cirrhosis, and radiotherapy-induced liver damage. Furthermore, it is commonly prescribed as an adjunct therapy during prolonged illness and the recovery process [47]. The antioxidant properties of Liv-52 are widely recognized as one of the fundamental mechanisms underlying its hepatoprotective effects [48]. Liv-52 inhibits mitochondrial β-oxidation of fatty acids due to its capacity to reduce LPO, thereby decreasing MDA levels and preventing GSH depletion [18]. Various experimental studies conducted on animal models have reported that Liv-52 application reduces the oxidative stress environment in drug-induced liver injury and demonstrates significant hepatoprotective effects by lowering serum AST and ALT levels [45,49]. Indeed, Çimen et al. reported that Liv-52 significantly reduced both oxidative stress and histopathological damage in a liver ischemia/reperfusion model [48]. Similarly, our findings indicate that Liv-52 exerts protective effects against HCQ-induced liver damage. Liv-52 reduced the increase in hepatic MDA levels and significantly preserved tGSH, SOD, and CAT, supporting its role in preventing oxidative stress-related injury. Both Liv-52 and ATP enhanced antioxidant defense, though Liv-52 was more effective in lowering serum AST and ALT levels. Notably, the combination therapy led to greater improvement, with biochemical parameters approaching those of the healthy controls, and the lack of statistical differences in AST and ALT levels suggests substantial attenuation of hepatocyte damage.

Histopathological examinations of liver tissue yielded results consistent with the biochemical findings. In the group treated with HCQ, severe parenchymal destruction, hemorrhage, edema, and significant vascular congestion were observed. These findings align with histopathological data describing HCQ-induced hepatocellular damage in the literature [21]. While ATP and Liv-52 applications partially reduced histopathological damage, in the group where both agents were administered together, the liver tissue structure was found to be quite close to a healthy morphology, with minimal vascular congestion.

Our study has certain limitations. The dose–response relationship was not evaluated, and only an acute toxicity model was used. In addition, measuring ATP levels in the liver tissues of animals treated with Liv-52 could have contributed to a better understanding of its potential effects. Therefore, further studies investigating different doses, administration durations, and additional mechanisms are needed. The limited sample size in our study (n = 6) poses a restriction, particularly in the interpretation of small differences between groups. Therefore, the findings should be evaluated within this limitation. The absence of control groups treated with ATP or Liv-52 alone (in the absence of HCQ) is another limitation. This omission makes it difficult to determine whether these agents have independent effects on liver oxidation and histology. Since our initial aim was solely to investigate HCQ-induced liver injury and the potential protective effects of ATP and Liv-52, such control groups were not included in the study design. Another limitation is that the high-dose, short-duration HCQ regimen used in this study reflects an acute toxicity model and is restricted to animal experiments; therefore, the findings cannot be generalized to long-term or high-dose clinical treatment in humans and remain limited to a preclinical framework. Although we obtained important findings regarding the potential hepatoprotective effects of ATP, Liv-52, and their combination against HCQ-induced oxidative liver injury, the absence of evaluations of key oxidative stress regulators such as Nrf2 and GST, as well as inflammatory markers including IL-6, IL-10, TNF-α, and NF-κB, represents a significant limitation. Future advanced studies will clearly elucidate the mechanisms through which ATP and Liv-52 exert their hepatoprotective effects. The underlying mechanisms of the hepatoprotective effects of ATP and Liv-52 could be more clearly elucidated through advanced molecular analyses such as Western blot and qRT-PCR. Evaluating the effects of these agents on other organs affected by HCQ toxicity may also provide a broader perspective. Additionally, dose–response studies are needed to determine whether the protective effects observed in the acute model persist over the long term.

## 4. Materials and Methods

### 4.1. Animals

The experimental study was conducted using 30 male albino Wistar-type rats, with body weights ranging from 270 to 281 g and aged 9–10 weeks. The animals were procured from the Experimental Animals Application and Research Center of Erzincan Binali Yıldırım University. The animals were allocated into five groups at random, with each group exhibiting a similar mean body weight. Before the commencement of the experiment, the rats were kept in groups of six within standard laboratory wire cages (20 cm high, 35 cm wide, 55 cm long; floor area: 1925 cm^2^) to allow for acclimatization to the laboratory conditions. The housing environment was maintained on a 12 h light/12 h dark cycle, at a constant temperature of 22 °C, with relative humidity ranging from 30% to 70%. Animals were granted free access to tap water and a commercially available pelleted feed (laboratory animal chow; Bayramoglu Stock Company, Erzurum, Turkey) throughout the study. Experimental protocols adhered to the European Parliament and Council Directive 2010/63/EU (Approval ID: 2016-24-199) [50] and were carried out in line with the ARRIVE reporting standards [51]. All experimental procedures involving animals were performed in the laboratories of the Experimental Animal Application and Research center at Erzincan Binali Yıldırım University.

### 4.2. Chemicals

The hydroxychloroquine (Plaquenil^®^ 200 mg tablet) used in this study was obtained from Sanofi (Istanbul, Turkey), thiopental sodium (Pental Sodyum^®^ 0.5 g vial) from IE Ulagay (Istanbul, Turkey), ATP (10 mg/mL vial) was supplied by Zdorove Narodu (Kharkiv, Ukraine), and Liv-52 (Liv.52^®^ tablet) was sourced from Himalaya Wellness Company (Bangalore, Karnataka, India).

### 4.3. Experimental Design and Grouping

#### 4.3.1. Experimental Design

The sample size was determined to utilize the smallest number of animals permissible in accordance with the 4R requirements [52]. Criteria including slumped posture, diminished mobility, and injuries inflicted by other animals were employed to remove subjects throughout the experiment and data points during analysis. No exclusions occurred throughout the experiment. The random number table was utilized to generate the randomization sequence. Cages and animals are assigned numbers to reduce potential confounding variables.

#### 4.3.2. Experimental Groups

The animals were randomly assigned to five experimental groups: healthy control (C), hydroxychloroquine only (H), ATP combined with HCQ (AH), Liv-52 combined with HCQ (LH), and a combination of ATP, Liv-52, and HCQ (ALH).

### 4.4. Experiment Procedure

The ATHQG group (n = 6) received 4 mg/kg of ATP intraperitoneally (IP). The LVHQG group (n = 6) was given 20 mg/kg of Liv-52 orally via an oral gavage into the stomach [48]. The ALH group (n = 6) received ATP + LIV-52 in the specified doses using the same method. Animals in the C (n = 6) and H (n = 6) groups administered an equivalent volume of distilled water as a solvent. One hour after receiving ATP, Liv-52 and distilled water, all of the rats except those in the C group were given 120 mg/kg of HCQ orally via an oral gavage into their stomachs, twice daily. The acute dose of 120 mg/kg used for HCQ administration was selected based on previous literature studies [21,53]. ATP and Liv-52 were used once daily. This procedure was repeated for a duration of one week. At the end of this period, all animals in the groups were sacrificed under high-dose thiopental sodium (50 mg/kg) anesthesia, and liver tissues were extracted. The levels of MDA, tGSH, SOD, and CAT were measured in the extracted liver tissues. ALT and AST activities were assessed in blood samples taken from the tail veins. The tissues were also examined histopathologically. The results obtained from all experimental groups were evaluated by comparison between groups.

### 4.5. Biochemical Analysis

#### 4.5.1. Preparation of Samples

Approximately 1 g of liver tissue was excised from each rat and immediately rinsed with 0.9% sodium chloride solution to remove blood and debris. The samples were then homogenized under ice-cold conditions using a high-speed tissue homogenizer. Following homogenization, 2 mL of 1.15% potassium chloride (KCl) buffer (pH 7.4) was added to each sample. The homogenates were centrifuged at 10,000× *g* for 15 min at 4 °C, and the supernatants were collected for subsequent biochemical analyses. Levels of MDA and tGSH, as well as the enzymatic activities of SOD and CAT, were determined from the supernatant fractions. To ensure intergroup comparability, all biochemical values were normalized to total protein content and expressed as milligrams per gram of protein (mg/g protein).

#### 4.5.2. Determination of MDA, tGSH, SOD, CAT and Protein in Liver Tissue

The levels of MDA, GSH, and SOD in tissue samples were quantified using the protocols provided with their respective rat ELISA kits: MDA (Product No. 10009055), total GSH (Product No. 703002), and SOD (Product No. 706002), all from Cayman Chemical Co. (Ann Arbor, MI, USA). CAT activity was determined according to the method described by Goth [54]. Protein concentrations were determined using the Bradford assay, which relies on the binding of Coomassie Brilliant Blue G-250 dye to proteins. The absorbance of the resulting complex was spectrophotometrically measured at 595 nm [55].

#### 4.5.3. Determination of Blood Serum ALT and AST

Venous blood samples were collected into sterile tubes without anticoagulant. After complete clot formation, the serum was separated by centrifugation and stored at –80 °C for subsequent analyses. The activities of serum ALT and AST were determined spectrophotometrically as indicators of liver function using the Cobas 8000 automated analyzer (Roche Diagnostics GmbH, Mannheim, Germany) and commercially available assay kits (Roche Diagnostics GmbH, Mannheim, Germany), in accordance with the manufacturer’s instructions.

### 4.6. Histopathological Analysis

Tissue samples from the subjects were fixed in 10% formaldehyde solution for a duration of 72 h. After fixation, tissue samples were embedded in cassettes and rinsed under running water for 24 h, followed by dehydration through ascending concentrations of ethanol (70%, 80%, 90% and 100%) to remove residual water. Liver tissues rendered transparent with xylol were embedded in paraffin blocks, and sections of 4–5 μm thickness were obtained using a microtome. The obtained sections were stained using Hematoxylin-eosin (H&E) and subsequently examined and photographed using the Olympus DP2-SAL firmware program (Olympus^®^ Inc., Tokyo, Japan). A blinded pathologist conducted the histopathological assessment for the study groups to ensure objective evaluation. Histopathological examination of liver tissue was performed for signs of parenchymal destruction, hemorrhage, interstitial edema, and vascular dilatation or congestion. All histopathological findings were assessed using a semi-quantitative scoring system: 0 = absent, 1 = mild, 2 = moderate and 3 = severe.

### 4.7. Statistical Analysis

All statistical analyses were performed using IBM SPSS Statistics for Windows, version 27.0 (IBM Corp., Armonk, NY, USA, 2020). Figure 1, Figure 2, Figure 3 and Figure 4 were generated using GraphPad Prism 8.0.1 (GraphPad Software, San Diego, CA, USA, 2018). All biochemical data are presented as mean ± standard deviation. The assumption of normality was assessed using the Shapiro–Wilk test. To evaluate mean differences between groups, one-way ANOVA or Welch’s ANOVA was used when the normality assumption was met. When normality was satisfied, the homogeneity of variances assumption was assessed using Levene’s test. Pairwise comparisons were performed using Tukey’s honestly significant difference (HSD) post hoc test following one-way ANOVA when homogeneity of variances was confirmed, or using the Games–Howell post hoc test following Welch’s ANOVA when the assumption was violated. Histopathological data are expressed as medians with corresponding ranges (minimum–maximum). To evaluate differences among groups for histopathological outcomes, the Kruskal–Wallis test was applied; when significant, Dunn’s test with Bonferroni correction was used for pairwise comparisons, and adjusted *p*-values are presented. The findings were considered statistically significant when *p*-values were below 0.05.

## 5. Conclusions

The effects of HCQ on liver tissue in rats were investigated using biochemical and histopathological methods. Our experimental results indicate that oxidative stress developed in the liver tissue of animals treated with HCQ. It was demonstrated that both ATP and Liv-52, administered separately and in combination, provided significant protective effects against HCQ-induced hepatotoxicity. The combination treatment was observed to exhibit the most pronounced protective effect; it significantly reduced MDA levels, improved GSH, SOD, and CAT levels more effectively, and produced significant decreases in AST and ALT values (*p* < 0.05). The best level of recovery from HCQ-induced tissue damage was observed in the ALH group, where ATP and Liv-52 were administered in combination. In conclusion, the findings suggest that ATP and Liv-52 may exert a potential protective effect in reducing acute HCQ-induced liver injury in a preclinical context.

## Figures and Tables

**Table 1:** Quantitative assessment of histopathological changes in rat liver tissue.

Groups	Histopathological Grading Data			
	Parenchymal Destruction	Hemorrhage	Edema	Vascular Dilatation/Congestion
C	0.00 (0.00–0.00)	0.00 (0.00–0.00)	0.00 (0.00–0.00)	0.00 (0.00–0.00)
H	3.00 *** (2.00–3.00)	3.00 *** (2.00–3.00)	3.00 *** (3.00–3.00)	3.00 *** (2.00–3.00)
AH	2.00 * (1.00–2.00)	2.00 * (1.00–3.00)	1.50 * (1.00–2.00)	2.00 * (1.00–2.00)
LH	1.50 * (1.00–2.00)	2.00 ** (2.00–3.00)	1.50 * (1.00–2.00)	1.50 * (1.00–2.00)
ALH	1.00 +++ (0.00–1.00)	1.00 +++ (0.00–2.00)	1.00 +++ (0.00–1.00)	1.00 +++ (0.00–2.00)
Group comparisons	***p*-values**			
C vs. H	<0.001	<0.001	<0.001	<0.001
C vs. AH	0.030	0.037	0.067	0.058
C vs. LH	0.079	0.015	0.067	0.142
C vs. ALH	1.000	1.000	1.000	0.588
H vs. AH	1.000	1.000	0.358	0.588
H vs. LH	0.709	1.000	0.358	0.281
H vs. ALH	0.009	0.019	0.011	0.058
AH vs. LH	1.000	1.000	1.000	1.000
AH vs. ALH	0.709	1.000	1.000	1.000
LH vs. ALH	1.000	0.603	1.000	1.000
K-W	23.673	23.692	25.026	22.376
df	4	4	4	4
Asymptotic Sig.	<0.001	<0.001	<0.001	<0.001

Values are expressed as median (min–max). * *p* < 0.05 vs. C, ** *p* < 0.01 vs. C, *** *p* < 0.001 vs. C. +++ *p* < 0.001 vs. H. n = 6 for all groups. Statistical analysis: Kruskal–Wallis test followed by Dunn’s post hoc test. Abbreviations: ATP: adenosine triphosphate; HCQ: hydroxychloroquine; C: healthy group; H: hydroxychloroquine alone group; AH: ATP + HCQ group; LH: Liv-52 + HCQ group; ALH: ATP + Liv-52 + HCQ group; df: degree of freedom.

## Data Availability

Data from the research can be obtained from the corresponding author upon reasonable request.

## Abbreviations

The following abbreviations are used in this manuscript:

HCQ	Hydroxychloroquine
MDA	Malondialdehyde
tGSH	Total Glutathione
SOD	Superoxid Dismutase
CAT	Catalase
ATP	Adenosine Triphosphate
ALT	Alanine Aminotransferase
AST	Aspartate Aminotransferase
ROS	Reactive Oxygen Species
ELISA	Enzyme-linked Immunosorbent Assay
LPO	Lipid Peroxidation
